# Results of primary versus recurrent surgery to treat stress urinary incontinence in women

**DOI:** 10.1007/s00192-015-2627-7

**Published:** 2015-03-10

**Authors:** Maarten J. van der Doelen, Mariëlla I. J. Withagen, Mark E. Vierhout, John P. F. A. Heesakkers

**Affiliations:** 1 Department of Urology, Radboud University Medical Centre, Geert Grooteplein Zuid 10 (610), P.O. Box 9101, 6500 HB Nijmegen, The Netherlands; 2 Department of Obstetrics and Gynecology, University Medical Center Utrecht, Utrecht, The Netherlands; 3 Department of Obstetrics and Gynecology, Radboud University Medical Centre, Nijmegen, The Netherlands

**Keywords:** Recurrence, Reoperation, Suburethral slings, Urinary incontinence, Stress

## Abstract

**Introduction and hypothesis:**

We compared cure rates and complication rates in patients who had undergone primary or recurrent (secondary) surgery for stress urinary incontinence (SUI).

**Methods:**

A retrospective cohort study that included patients who underwent surgery to treat SUI in a tertiary referral center was carried out. All patients had, predominantly, SUI. Exclusion criteria were patients with a neurogenic bladder or a neobladder and patients without postoperative follow-up (FU). The primary objective was to assess the success rate, defined as cured SUI or improved SUI at six weeks and at the latest available moment of FU. The secondary objective was to assess complications.

**Results:**

A total of 541 women with SUI underwent surgery for SUI between 2002 and 2010. After exclusion of 102 patients a total of 242 patients with primary SUI and 197 patients with recurrent SUI were identified. The success rate at first FU was 89 %. At last FU (median 205 days) the success rate was 83 % (*P* < 0.01). There were no significant differences in success rate between primary and recurrent surgery at first FU. The overall success rate of primary surgery was 86 %; for recurrent surgery it was 79 %. During surgery, 27 bladder injuries occurred (6.2 %), with no significant difference between the two groups. At last FU, 11 patients (2.6 %) had persistent residual volume, necessitating prolonged clean intermittent self-catheterization.

**Conclusions:**

The results of recurrent surgery to treat recurrent or persistent SUI are still good in experienced hands and do not significantly differ from results of primary surgery. The complication rates are comparable to those of primary surgery.

## Introduction

Stress urinary incontinence (SUI) is the complaint of involuntary loss of urine on physical exertion, or on sneezing or coughing [1]. Nowadays, there are plenty of treatments for SUI. The mid-urethral sling (MUS) is currently the gold standard treatment for SUI, with high success rates of approximately 77–85 % [2, 3].

Nonetheless, a subgroup of patients experience recurrent or persistent SUI after treatment of their complaints and request retreatment. An estimated 8–17 % of the women who have undergone surgical therapy for urinary incontinence, are likely to undergo a second surgical treatment within 8–10 years [4, 5].

There are several options for the management of recurrent SUI, including repeating the previously performed procedure, MUS, Burch colposuspension, and bulking agent injections [6, 7]. Unfortunately, performing a recurrent procedure attempting to solve SUI does not guarantee complete dryness.

To date, there have been limited data on the optimal approach to recurrent SUI, in particular for recurrent SUI after more than two surgical procedures. The aim of this study was to compare cure rates and complication rates in patients who had undergone primary and recurrent surgery for SUI.

## Materials and methods

### Patient selection

We conducted a retrospective cohort study including all medical records of the patients who underwent surgical SUI treatment between January 2002 and March 2010 in the departments of gynecology and urology of the Radboud University Medical Centre, a tertiary referral center in the Netherlands.

**Fig. 1:**
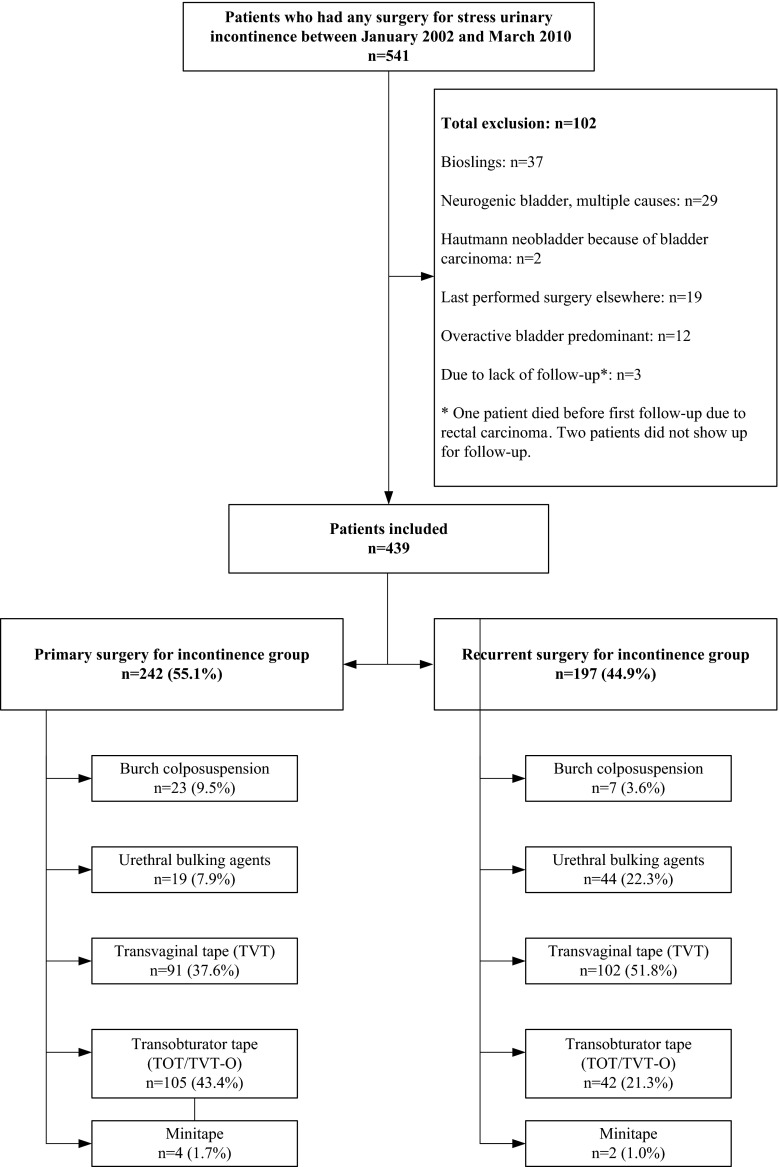
Patient enrolment

Patient enrolment is shown in Fig. 1. A total of 541 patients were identified. All patients had, predominantly, SUI. SUI was defined as the complaint of involuntary loss of urine on physical exertion, or on sneezing or coughing [1]. Exclusion criteria were patients with predominantly overactive bladder (OAB) symptoms (*n* = 12), neurogenic bladder of various causes (*n* = 29), patients with a neobladder (*n* = 2), patients who had last undergone surgery elsewhere (*n* = 19), and patients without postoperative follow-up (*n* = 3). Patients who had undergone a xenograft sling (*n* = 37) were excluded because of the experimental setting of the surgery in a research setting.

Every patient was added to our database only once, even if the patient underwent surgery in our hospital more than once. In recurrent cases, only the result of the last surgical procedure was analyzed.

### Study outcomes

The primary outcome of the study was the cure rate based on findings during physical assessment and patient statements at follow-up at six weeks and at the latest available follow-up. Subjective cure/improvement was assessed by an open question during clinical visits and a standardized questionnaire. Objective cure/improvement was tested by a cough test with a full bladder, voiding diary or micturition diary, and a urodynamic investigation if indicated. SUI was considered to have been cured when episodes of involuntary urine leakage during stressful activities according to the patient remained absent and there was no demonstrable SUI during physical examination. Improvement was defined as a significant decrease in urine leakage without further treatment. Failure to cure SUI was classified as unchanged or deteriorated leakage of urine. An unchanged condition was defined as persistent SUI after surgery for SUI. If the urine leakage due to SUI worsened according to the patient, this was classified as deterioration.

Secondary outcomes included the number of peri-operative and postoperative complications, including vaginal tape exposure, pain, and longer term voiding dysfunction.

### Surgical procedures, postoperative care, and follow-up

Possible surgical procedures were Burch colposuspension, tension-free vaginal tape (TVT), transobturator tape (TOT), minitape, and periurethral bulking agents.

Operative time and blood loss were measured in every surgical procedure for SUI. Substantial blood loss was defined as more than 500 ml.

If another tape had been placed previously, the tape placed during the primary operation was not specifically identified or removed if it was still in place and not hampering the secondary procedure. Intraoperative urethrocystoscopy was performed during all TVT and minitape operations to ensure that no damage had occurred to the urethra or bladder during the procedure.

Among the patients who received a TVT, a TOT or a minitape, a transurethral catheter (TUC) was inserted and kept in position for at least one day.

When performing a Burch colposuspension, a suprapubic catheter (SPC) was inserted in every patient during surgery under direct visualization by intraoperative cystoscopy. Voiding attempts were started one day postoperatively after TVT, TOT, and minitape surgery, and two days postoperatively after Burch colposuspension. If the patient was able to void well and the postvoid residual volume was less than 100 ml on two consecutive occasions, the SPC or TUC was removed and the patient was discharged. In the case of unsatisfactorily voiding, clean intermittent self-catheterization (CISC) was started or the patient was discharged with a TUC or SPC in situ and checked after seven days. If the patient was unable to void well at that time, the catheter was kept in place for another week or the patient started CISC and was regularly checked in our hospital.

The periurethral bulking injections were given with local anesthesia and injected into the submucosa through the urethra under direct urethroscopic guidance. Three to four deposits were placed at positions 0.5–1 cm distal to the bladder neck. Patients were discharged after successful voiding without significant postvoid residual urine (less than 100 ml).

Patients were assessed at approximately six weeks and three months postoperatively, and thereafter if necessary. Median time between surgery and the last follow-up was 205 days.

The cure rate was assessed by the observation of involuntary leakage from the urethra synchronous with coughing and also based on patient statements.

### Statistical analysis

In accordance with Dutch law, retrospective observational studies are exempt from submission for approval to a medical ethics review committee. However, the principles of the Declaration of Helsinki were followed. We used the Mann–Whitney *U* test for independent variables without a normal distribution and the Chi-squared test for categorical variables.

All statistical analyses were performed using SPSS software for Windows (version 17.0; SPSS, Chicago, IL, USA), with *P* values < 0.05 considered statistically significant.

## Results

### Patient population

A total of 439 patients met the inclusion criteria, of whom 242 had had primary anti-incontinence surgery and 197 recurrent surgery.

Of the 439 patients included, 40 % (*n* = 176) were treated by gynecologists and 60 % (*n* = 263) were treated by urologists. Urologists treated 85 % (*n* = 167) of the recurrent SUI cases.

### Baseline characteristics

Patient characteristics are listed in Table 1. Patients with recurrent SUI symptoms had more frequent voiding difficulties and used TUC or performed CISC more frequently (1.2 % vs 8.1 %; *P* < 0.01).

**Table 1:** Baseline characteristics

	Complete cohort (*N* = 439)			Primary surgery group (*n* = 242)			Recurrent surgery group (*n* = 197)			*P*
	Number	Median [range] or mean ± SD or number (%)		Number	Median [range] or mean ± SD or number (%)		Number	Median [range] or mean ± SD or number (%)		
Age at time of surgery, years	439	59	[22–94]	242	56	[38–94]	197	62	[22–92]	<0.01*
Parity, number	361	2	[0–6]	217	2	[(0–6]	144	2	[0–6]	0.16*
Menopause and/or using HRT	388	250	(64.4)	213	115	(54.0)	175	135	77.1	<0.01**
BMI, kg/m^2^	426	27.2	±5.0	235	26.7	±4.8	191	27.9	±5.1	0.03*
Smoking	421	84	(19.1)	231	46	(19.9)	190	38	(20.0)	0.98**
Current respiratory disease	439	81	(18.5)	242	41	(16.9)	197	40	(20.3)	0.37**
Surgical history										
POP surgery										
≥ 1 surgery for POP	439	94	(21.4)	242	34	(14.0)	197	60	(30.5)	<0.01**
≥ 2 surgeries for POP	439	25	(5.7)	242	7	(2.9)	197	18	(9.1)	0.01**
SUI surgery										
≥ 1 surgery for SUI	439	197	(44.9)	242	–	–	197	197	(100.0)	–
≥ 2 surgeries for SUI	439	97	(22.1)	242	–	–	197	97	(49.2)	–
≥ 3 surgeries for SUI	439	38	(8.7)	242	–	–	197	38	(19.3)	–
Hysterectomy	439	140	(31.9)	242	59	(24.4)	197	81	(41.1)	<0.01**
AH and SVH	433	87	(20.1)	241	32	(13.3)	192	55	(28.6)	<0.01**
VH	433	48	(11.1)	241	26	(10.8)	192	22	(11.5)	0.83**
MUI	437	190	(43.5)	241	99	(41.1)	196	91	(46.4)	0.26**
Micturition frequency										
Daily frequency	393	8	[3–20]	217	8	[4–20]	176	8	[3–17]	0.84*
Nightly frequency	399	1	[0–6]	222	1	[0–5]	177	2	[0–6]	0.22*
Recurrent UTI	429	104	(24.2)	235	45	(19.1)	194	59	(30.4)	<0.01**
Preoperative use of a CISC or TUC	439	19	(4.3)	242	3	(1.2)	197	16	(8.1)	<0.01**

*HRT* hormone replacement therapy, *BMI* body mass index, *SUI* stress urinary incontinence, *AH* abdominal hysterectomy, *SVH* supravaginal hysterectomy, *VH* vaginal hysterectomy, *MUI* mixed urinary incontinence, *UTI* urinary tract infection, *CISC* clean intermittent self-catheterization, *TUC* transurethral catheter

*Calculated using the Mann–Whitney *U* test

**Calculated using the Chi-squared test

### Peri-operative outcomes

Peri-operative data are listed in Table 2.

**Table 2:** Peri-operative data and complications

	Complete cohort (*N* = 439)			Primary surgery group (*n* = 242)			Recurrent surgery group (*n* = 197)			*P*
	Number	Median [p5–p95] or number (%)		Number	Median [p5–p95] or number (%)		Number	Median [p5–p95] or number (%)		
Concomitant surgery	439	129	(29.4)	242	67	(27.7)	197	62	(31.5)	0.39*
Operating time (skin to skin), min^a^	286	25	[9–49]	160	25	[11–47]	127	25	[6–50]	0.58**
Burch colposuspension	5	57	[50–65]	4	58	[50–65]	1	57	[57–57]	1.00**
Urethral bulking agents	43	11	[4–27]	9	12	[9–20]	34	10	[4–30]	0.45**
TVT	130	28	[19–50]	68	27	[18–46]	62	29	[18–53]	0.67**
TVT-O/TOT	103	22	[11–39]	74	22	[11–39]	29	28	[15–58]	0.03**
Minitape	5	22	[17–24]	4	23	[17–24]	1	20	[20–20]	0.47**
Blood loss (ml)^a^	265	50	[0–200]	145	50	[0–200]	120	50	[0–200]	0.08**
Burch colposuspension	4	150	[50–600]	3	150	[50–150]	1	600	[600–600]	0.16**
Urethral bulking agents	58	0	[0–0]	18	0	[0–0]	40	0	[0–0]	0.50**
(TVT)	106	50	[10–265]	54	50	[10–350]	52	50	[10–200]	0.04**
TVT-O/TOT	96	50	[10–215]	70	50	[10–145]	26	50	[10–365]	0.67**
Minitape	1	50	[50–50]	0	–	–	1	50	[50–50]	–***
Perioperative complications										
Bladder lesion	439	27	(6.2)	242	11	(4.5)	197	16	(8.1)	0.12*
Urethral lesion	439	1	(0.25)	242	0	(0.0)	197	1	(0.5)	–***
Blood loss > 500 ml^a^	310	17	(5.5)	175	10	(5.7)	135	7	(5.2)	0.84*

*TVT* transvaginal tape, *TVT-O/TOT* transobturator tape

*Calculated using the Chi-squared test

**Calculated with Mann–Whitney *U* test

***Not enough valid cases to calculate a *P* value

^a^Without concomitant surgery

In 29 % of cases the incontinence surgery was combined with other surgery, such as pelvic organ prolapse (POP) surgery or removal of previous tapes. After the exclusion of concomitant surgeries, we found seven cases (5.2 %) of excessive bleeding during recurrent procedures carried out to treat SUI and 10 (5.7 %) during primary procedures.

One urethral lesion was observed in a patient with an incorrect location of her previous TVT sling, which was removed during the same surgery. The integrity of the urethra was restored and the patient received long-term indwelling catheterization.

All 27 (6.2 %) bladder injuries occurred during TVT surgery; however, there was no significant difference between the two groups. All bladder lesions were recognized intraoperatively by cystoscopy. If a bladder lesion was noticed, the sling was removed, the trocar was replaced and the sling reinserted. There was no prolonged hospital stay in these patients.

### Postoperative outcomes

Postoperative data and long-term data are listed in Table 3.

**Table 3:** Postoperative data and long-term data

	Complete cohort (*N* = 439)			Primary surgery group (*n* = 242)			Recurrent surgery group (*n* = 197)			*P*
	Number	Median [range] or number (%)		Number	Median [range] or number (%)		Number	Median [range] or number (%)		
Postoperative data										
Pain ^a^	439	22	(5.0)	22	16	(72.7)	22	6	(27.3)	0.09*
Pain ^b^	310	7	(2.3)	175	3	(1.7)	135	4	(3.0)	0.47**
After Burch colposuspension	5	2	(40.0)	4	2	(50.0)	1	0	(0.0)	1.00**
After TVT	137	2	(1.5)	74	0	(0.0)	63	2	(3.2)	0.21**
After TOT	103	3	(2.9)	74	1	(1.4)	29	2	(6.9)	0.19**
Hematoma ^a^	439	4	(0.9)	242	3	(1.2)	197	1	(0.5)	0.63**
Re-intervention due to complication ^a^	439	4	(0.9)	242	3	(1.2)	197	1	(0.5)	0.63**
Urinary retention ^c^	420	53	(12.6)	239	31	(13.0)	181	22	(12.2)	0.80*
Temporary	420	42	(10.0)	239	25	(10.5)	181	17	(9.4)	0.77*
Persistent	420	11	(2.6)	239	6	(2.5)	181	5	(2.8)	0.77*
Duration of urinary catheter (days) ^a^	420	1	[0–34]	236	1	[0–9]	184	1	[0–34]	0.10***
Hospital stay (days) ^a^	439	1	[0–15]	242	1	[0–10]	197	1	[0–15]	<0.01***
Hospital stay (days) ^b^	310	1	[0–15]	175	1	[0–8]	135	1	[0–15]	0.14***
Long-term data										
Micturition frequency ^a^										
Daily frequency	191	8	[2–20]	105	8	[2–20]	86	8	[3–20]	0.31***
Nightly frequency	187	2	[0–7]	103	1	[0–7]	84	2	[0–5]	0.43***
De novo OAB symptoms ^a^	243	52	(21.4)	139	24	(17.3)	104	28	(26.9)	0.07*
Pain ^a^	431	57	(13.2)	236	38	(16.1)	195	19	(9.7)	0.05*
Pain ^b^	308	32	(10.3)	174	21	(12.1)	134	11	(8.1)	0.27*
After TVT	136	16	(11.8)	74	9	(12.2)	62	7	(11.3)	0.87*
After TOT	102	11	(10.8)	73	8	(11.0)	29	3	(10.3)	0.93*
Vaginal exposure ^a^	439	13	(3.0)	242	8	(3.3)	197	5	(2.5)	0.64*
After MUS	346	8	(2.3)	200	5	(2.5)	146	3	(2.1)	0.79**
After bulking agents	63	5	(7.9)	19	3	(15.8)	44	2	(4.5)	0.13**

*Calculated using the Chi-squared test

**Calculated using Fisher’s exact test

***Calculated using the Mann–Whitney *U* test

^a^Concomitant surgery included

^b^Concomitant surgery excluded

^c^Not including the patients who had pre-operative CIC, CAD or a suprapubic catheter (*n* = 19)

Postoperative complications before discharge were hematoma (*n* = 4), subsequent bleeding (*n* = 2), urinary tract infection during hospital stay (*n* = 8), and pain (*n* = 22). Re-interventions (*n* = 4) were needed in cases of peri-operative bleeding (*n* = 1), postoperative bleeding (*n* = 2), and retensioning of a TVT (*n* = 1). Postoperative pain was observed significantly more frequently in patients who received concomitant surgery (68 % vs 32 %; *P* < 0.01) and did not occur significantly more often in patients who underwent recurrent surgery (27 % vs 73 %; *P* = 0.09). Most reported postoperative complaints of pain were located inguinally after TOT (*n* = 11; 7 %) and suprapubically after Burch colposuspension (*n* = 7; 23 %). At first follow-up (six weeks postoperatively), 64 % (*n* = 14) of the 22 patients with postoperative pain did not have pain anymore. At last follow-up, 77 % (*n* = 17) of the 22 patients with postoperative pain no longer had pain.

Before discharge, 53 patients (12.6 %) were unable to void properly or had post-void residual of more than 100 ml. Most patients were able to void well after one week without postvoid residual. Eleven patients (2.6 %) had persistent retention, necessitating prolonged CISC or an indwelling catheter. Two women wanted lysis of the tape, which was performed. Also, three patients had an extended postoperative transurethral catheter for special reasons (one urethral lesion and two patients with voiding dysfunction after a bladder lesion), but could void normally afterwards.

Pre-operatively, 19 patients had a TUC or a SPC or performed CISC. Directly after surgery four of these patients still had urinary retention and needed to perform CISC. The other 15 patients were able to void spontaneously without considerable residual volume, but ten of them needed a catheter at last follow-up (205 days).

### Long-term outcomes

The success rates of primary and recurrent surgery are compared in Table 4.

**Table 4:** Success of primary surgery vs recurrent surgery for stress urinary incontinence

		Success at first follow-up (6 weeks)		*P* value primary surgery compared with recurrent surgery*	Success at last follow-up		*P* value primary surgery compared with recurrent surgery*
		Number (%)	Number (%)		Number (%)	Number (%)	
	Number	Primary surgery *n* = 242	Recurrent surgery *n* = 197		Primary surgery *n* = 242	Recurrent surgery *n* = 197	
Burch colposuspension	30	21 (91.3)	4 (57.1)	0.07	20 (87.0)	4 (57.1)	0.12
Urethral bulking agents	63	12 (63.2)	36 (81.8)	0.11	9 (47.4)	30 (68.2)	0.12
TVT	193	84 (92.3)	94 (92.2)	0.97	82 (90.1)	88 (86.3)	0.41
TVT-O/TOT	147	99 (94.3)	34 (81.0)	0.01	94 (89.5)	33 (78.6)	0.08
Minitape	6	4 (100.0)	2 (100.0)	1.00	4 (100.0)	1 (50.0)	0.33
Total	439	220 (90.9)	170 (86.3)	0.127	209 (86.4)	156 (79.2)	0.046

*Calculated using the Chi-squared test

Overall, 89 % of the patients had relief, either complete or improved, of SUI symptoms at first follow-up. At last follow-up the success rate was 83 %. There were no significant differences between primary and recurrent surgery at first-follow-up, but at last follow-up the success rate of recurrent surgery was lower (79 %) than the success rate of 86 % for primary surgery at last follow-up (*P* = 0.05).

Of the 100 patients in whom primary surgery failed, 68 patients had undergone primary surgery with a MUS previously. Most of them were treated with a new MUS (79 %, *n* = 54); the success rate was 85 %. Another 16 % (*n* = 11) were treated using periurethral bulking agents; the success rate was 73 %.

Twenty-three patients had undergone primary surgery using periurethral bulking agents previously. Most of them were treated with an MUS (78 %, *n* = 18); the success rate was 89 %. Another 17 % (*n* = 4) were treated using periurethral bulking agents; the success rate was 75 %.

At first follow-up, vaginal exposure was reported in five patients (2.1 %) after primary surgery and in three patients (1.5 %) after recurrent surgery (*P* = 0.74).

Urethral mucosal exposure occurred twice after bulking agents and six times after MUS.

At last follow-up, two of these patients had persistent exposure in the vagina and needed re-excision of their MUS (*n* = 1) and bulking agents (*n* = 1). Five other patients reported new exposure at the last follow-up (three times after bulking agents and twice after MUS), all patients underwent surgical excision.

## Discussion

This manuscript clearly illustrates that the success rates of primary and secondary procedures are acceptable, with no significantly higher rates of complications. In this study we found success rates of 86 % for primary cases and 79 % for recurrent cases. Overall, only 17 % of patients experienced no relief of SUI symptoms six months after surgery.

In a recent review, Pradhan et al. noted a subjective cure rate following MUS for recurrent SUI after any previous surgery of 79 % at follow-up of 30 ± 29 months [8]. This cure rate is slightly lower than our success rate of 84 % for surgery with MUS at last follow-up, but other criteria for success were applied in the various studies and the follow-up of our study is much shorter.

We observed a lower cure rate with TOT (79 %) than with the retropubic tape (86 %) for recurrent SUI. This is in line with the results described in the review by Pradhan et al., although they reported lower success rates (54 %) than in this study [8]. This could also have been caused by the relatively low number of our patients who underwent TOT to treat recurrent SUI. These results contradict the results of the systematic review by Agur et al., who described TOT as having similar patient-reported and objective cure/improvement rates compared with retropubic TVT in the surgical treatment of women with recurrent SUI [9].

In our study, treatment with bulking agents resulted more often in improvement than in complete dryness (54 % and 8 % respectively). In patients with recurrent SUI symptoms the results with bulking agents were slightly higher (59 % and 9 % respectively). These low percentages are consistent with those of a previous study, in which bulking agents after previous MUS showed a cure rate of 35 % for a median follow-up of 10 months [10]. However, patient satisfaction was relatively high (77 %) in this study after this minimally invasive procedure. This implies that bulking agents could play a role in secondary treatment to improve SUI symptoms after a previous failed procedure, especially when more invasive procedures are not an option.

As we performed only seven Burch colposuspensions in patients with recurrent SUI symptoms, it is difficult to generate proper conclusions.

Stav et al. have argued that de novo OAB symptoms are more common in patients who underwent a repeat MUS to treat persistent or recurrent SUI [2]. In our study, de novo OAB symptoms were seen in 52 (21 %) patients, with no significant difference between the two groups.

According to a systematic review, the incidence of de novo urgency after MUS to treat recurrent SUI is between 5.5 and 20 % [8]. A possible explanation for the higher incidence of 27 % (after any recurrent procedure) in our series is the high number of patients who had undergone more than two previous operations to treat SUI. According to Pradhan et al., the rate of urinary tract injuries is 8 % with retropubic sling procedures during repeat continence surgery, while it is 4 % during primary retropubic sling procedures [8]. These results are consistent with those we found in our study (8 % and 5 % respectively).

As described above, we found seven cases of excessive bleeding (2 %) during recurrent procedures carried out to treat SUI. According to the literature, the incidence of excessive bleeding during repeat SUI surgery is 2–7 % [8].

Postoperatively, 12 % of patients had symptoms of voiding dysfunction after recurrent surgery. At last follow-up, 11 patients (3 %) had persistent residual volumes, necessitating prolonged CISC. There was no significant difference between the patients who underwent primary surgery and the patients who underwent recurrent surgery. Pradhan et al. described the frequency of voiding dysfunction after recurrent surgeries as varying between 4 and 16 % [8].

In our study, success rates of urethral bulking agents and TVT turned out to have significantly worsened by time of the last follow-up visit compared with the first follow-up. Repeat continence surgery is often difficult as a result of retropubic scarring and adhesions with the distortion in anatomy from previous surgeries, which may increase the risks of intraoperative complications [11]. Amaye-Obu and Drutz found that the first surgical approach for the management of SUI is the best, because cure rates decline proportionately with the number of subsequent operations performed [12]. We also found this trend in our data.

The impact of this study is limited by its retrospective design and descriptive nature. It is, therefore, susceptible to recall and interpretation bias. Moreover, since data were collected retrospectively, 30 % of the patients had a follow-up of less than 3 months. Therefore, the postoperative follow-up may have been too short to find recurrences of SUI in some cases.

The fact that our academic hospital is a tertiary care center limits the generalizability of our conclusions. Some patients needed multiple procedures to achieve optimal relief of their SUI symptoms. In our study, nearly 9 % of the patients needed three or more procedures, with a maximum of as many as eight previous procedures (*n* = 1).

Despite these limitations, this study is strengthened by the large number of patients, by the fact that all operations were performed in one center by experienced surgeons using the same surgical techniques, and by the detailed data available from operative and medical records.

## Conclusion

Results of recurrent surgery to treat recurrent or persistent SUI are still good in experienced hands and complication rates are comparable to those of primary surgery. The treatment of choice should always be adapted to the specific patient needs, especially in recurrent cases. In our opinion recurrent SUI symptoms should only be treated with utmost skill and only by trained and experienced surgeons, preferably in a tertiary referral center. Patients should be informed about the potential risks of recurrent SUI surgery, in particular about exposure and voiding difficulties. Our results support the claims of the contemporary literature that retropubic midurethral slings are the first choice in the treatment of SUI symptoms after failure of a previous anti-incontinence operation. Long-term follow-up is important because of a time-dependent decline in cure rate.

## Abbreviations

AH: Abdominal hysterectomy
BMI: Body mass index
CISC: Clean intermittent self-catheterization
HRT: Hormone replacement therapy
MUI: Mixed urinary incontinence
MUS: Midurethral sling
OAB: Overactive bladder
POP: Pelvic organ prolapse
SPC: Suprapubic catheter
SUI: Stress urinary incontinence
SVH: Supravaginal hysterectomy
TOT: Transobturator tape
TUC: Transurethral catheter
TUI: Transurethral injection
TVT: Tension-free vaginal tape
UI: Urinary incontinence
UTI: Urinary tract infection
VH: Vaginal hysterectomy
